# The Application of the Intolerance of Uncertainty Model to Gambling Urge and Involvement

**DOI:** 10.3390/ijerph192214738

**Published:** 2022-11-09

**Authors:** Hui Zhou, Eva P. W. Hung, Li Xie, Zhen Yuan, Anise M. S. Wu

**Affiliations:** 1 Department of Psychology, Faculty of Social Sciences, University of Macau, Macao, China; 2 Centre for Cognitive and Brain Sciences, Institute of Collaborative Innovation, University of Macau, Macao, China; 3 Department of Social Science, The Hang Seng University of Hong Kong, Hong Kong, China; 4 School of Pre-School Education, Chongqing University of Education, Chongqing 400065, China; 5 Bioimaging Core, Faculty of Health Sciences, University of Macau, Macao, China

**Keywords:** intolerance of uncertainty, impulse control, limited access to emotion regulation strategies, gambling urge, gambling involvement, Chinese, gamblers

## Abstract

Background: Drawing on the intolerance of uncertainty model, this study aimed to examine whether intolerance of uncertainty and emotion regulation difficulties (in terms of impulse control difficulties and limited access to emotion regulation strategies) contributed to individual differences in gambling urge and involvement. Methods: Convenience sampling was used to recruit Chinese adult participants who had engaged in buying lottery tickets and other gambling activities in the past year. They were asked to complete an anonymous questionnaire survey, and a total of 580 valid cases (*M_age_* = 34.07, *SD* = 13.36; 50.4% female) were collected for data analysis. Results: Path analysis showed that the total effect of intolerance of uncertainty on gambling urge and involvement was significant and positive. However, only impulse control difficulties and not limited access to emotion regulation strategies fully mediated the effect of intolerance of uncertainty. Conclusions: As the first study to apply the intolerance of uncertainty model to real-life gambling, it found that individuals’ intolerance of uncertainty and impulse control difficulties contributed to more gambling urge and involvement. Improving emotion regulation skill (e.g., impulse control training) may, therefore, be considered in programs for promoting responsible gambling among Chinese gamblers.

## 1. Introduction

While gambling is commonly perceived as a form of recreational activity as individuals wager on something with uncertain outcome hoping to win valuable things, it can develop into a pathological condition, i.e., gambling disorder (GD), if individuals fail to control their gambling behaviors, leading to clinically significant impairments or distress [1]. The prevalence of GD ranges from 0.12% to 5.8% across countries and is particularly high among Chinese people [2]. Among the various gambling activities Chinese people engaged in, such as mahjong, pokers and casino games [3], buying lottery tickets is the most popular with an annual gross revenue of 373.285 billion CNY (approximately 54.09 billion USD) in 2021 [4]. Considering the potential threat of gambling to public health [3,5], this study applied the intolerance of uncertainty model [6] to understand individual differences in gambling urge and involvement through an examination of the antecedent roles of intolerance of uncertainty and emotion regulation difficulties.

### 1.1. Intolerance of Uncertainty and Gambling

Intolerance of uncertainty is an individual’s stable disposition to consider ambiguous and uncertain situations as threatening and distressing, which results in cognitive and behavioral attempts to avoid these events [7,8]. According to the intolerance of uncertainty model, which is a transdiagnostic psychological models of emotional disorders [9,10,11], people with a high level of intolerance of uncertainty are presumed to either ruminate the worst situation to prepare for the possible negative events or make efforts to dispel and avoid anxious feelings and thoughts caused by unpredictable events [6,12]. Regardless of their cognitive and behavioral strategies, they tend to be overly worried and anxious [13,14] and more susceptible to anxiety-related pathologies such as generalized anxiety disorder and obsessive-compulsive disorder [15,16]. 

Previous studies suggested that people resort to gambling to regulate and/or escape from negative emotions [17,18], but its relationship with intolerance of uncertainty was rarely examined. The only exceptions were two experimental studies which used the Iowa Gambling Task as a tool to assess impulsive decision making, and they demonstrated the preference for immediate but less valuable rewards rather than high-probability delayed rewards in individuals with a higher level of intolerance of uncertainty [19,20]. These findings suggested that people who are more intolerant to uncertainty are inclined to addictive behaviors which often bring immediate pleasure. Indeed, previous studies reported a positive association between intolerance of uncertainty and problematic Internet/smartphone use [21,22].

To fill the aforementioned research gap, the present study aimed to empirically examine whether intolerance of uncertainty is associated with gambling urge, defined as a psychological state involving a need, want, or desire to gamble [23], as well as gambling involvement in terms of frequency, expenditure, and knowledge acquisition. Based on the intolerance of uncertainty model, we hypothesized positive correlations between intolerance of uncertainty and gambling urge (Hypothesis 1), as well as between intolerance of uncertainty and those behavioral indicators of gambling involvement (Hypothesis 2).

### 1.2. The Roles of Emotion Regulation Difficulties

According to the intolerance of uncertainty model, intolerance of uncertainty also contributes to anxiety and related behaviors through promoting negative perceptions and expectations regarding one’s problems and problem-solving abilities [6,15]. These negative perceptions and expectations drive him/her to react negatively to existing problems and, hence, further increase his/her difficulties in regulating negative emotions (e.g., anxiety) and impulses [24,25]. As such, the positive correlation between intolerance of uncertainty and emotion regulation difficulties reported in previous research provided further empirical support to the theory [26,27,28,29].

Among the sub-factors of emotion regulation difficulties [30], both impulse control difficulties (reflecting the extent to which one can remain in control of his/her behaviors when feeling distressed) and limited access to emotion regulation strategies (reflecting the extent to which one can do to regulate his/her emotions once upset) are generally regarded as the direct and common consequences of intolerance of uncertainty [12,31,32]. Intolerance of uncertainty may contribute to both impulse control difficulties and limited access to emotion regulation strategies via increasing emotion regulation demand and decreasing cognitive resources. On one hand, individuals with a high level of intolerance of uncertainty interpret ambiguity as threatening [14] and, therefore, are prone to overwhelming worry [13,33], which leads to an increased need for emotion regulation in general. On the other hand, struggling with thoughts regarding uncertain events may deplete people’s cognitive resources. For example, people with a high level of intolerance of uncertainty are inclined to allocate unnecessary attention to non-target stimuli (e.g., distractors denoting spatial and temporal uncertainty) in experimental tasks [34], demonstrating an attentional bias due to intolerance of uncertainty. Such cognitive bias to uncertain events in life drains people’s finite cognitive resources [35,36], and thus less cognitive resources are available for controlling impulsive acts and developing and/or adopting strategies to reduce negative emotions. It is, therefore, not surprising that intolerance of uncertainty is positively associated with impulse control difficulties and limited access to emotion regulation strategies in previous studies [12,26,27]. Based on this, we hypothesized a positive association between intolerance of uncertainty and difficulties in emotion regulation in terms of both impulse control difficulties and limited access to emotion regulation strategies (Hypothesis 3).

As a potential consequence of intolerance of uncertainty, emotion regulation difficulties, in turn, may increase the probability of gambling behaviors or even GD. Previous studies have consistently reported the positive correlation between emotion regulation difficulties and disordered gambling levels [37,38,39], while disordered gamblers also reported more difficulties in emotion regulation than their healthy counterparts [40]. As gambling is associated with the expectation of gaining positive emotional experience and reducing boredom and distress across age groups and cultures [41,42,43], it has long been used as a means to regulate emotional states by gamblers [17,18,43]. Gamblers with emotion regulation problems or difficulties may, particularly, find it hard to refuse gambling during emotional occasions, and such low refusal efficacy is correlated with a higher gambling intention [44]. Therefore, we hypothesized that both impulse control difficulties and limited access to emotion regulation strategies would be positively correlated with both gambling urge (Hypothesis 4) and gambling involvement (Hypothesis 5).

**Figure 1:**
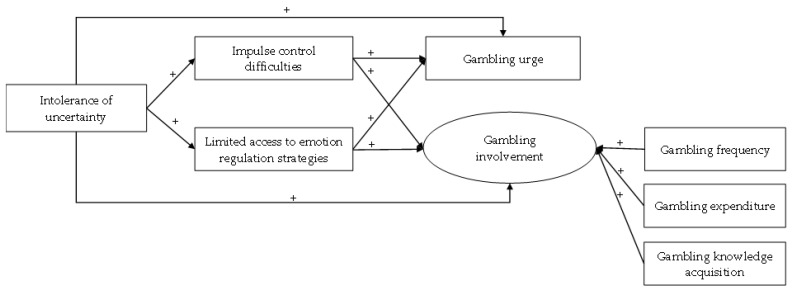
The hypothesized path model.

According to the intolerance of uncertainty model, people who are intolerant to uncertainty are vulnerable to maladaptive cognitive, emotional, and behavioral strategies to cope with uncertainty-related anxiety. Consistent with the model, both cognitive-emotional problems (i.e., impulse control difficulties and limited access to emotion regulation strategies) and addictive behaviors (i.e., gambling) are potential outcomes of high levels of intolerance of uncertainty. Furthermore, impulse control difficulties and limited access to emotion regulation strategies are potential mediators on the relationship between intolerance of uncertainty and addictive behaviors, given that the latter (e.g., gambling) is commonly perceived as a convenient means for emotion regulation by users [43,45]. A higher level of intolerance of uncertainty is likely to drive gamblers to experience more emotion regulation difficulties and more gambling behaviors. To our best knowledge, no published study has examined whether emotion regulation difficulties would mediate the relationship between intolerance of uncertainty and gambling (e.g., gambling urge and involvement in our study), whereas the mediating role of emotion regulation difficulties in the association between individuals’ negative life experience and GD has been suggested in a recent study [39]. The present study is the first to extend the model of intolerance of uncertainty to gambling and hypothesized that the two domains of difficulties in emotion regulation (impulse control difficulties and limited access to emotion regulation strategies) would bridge the association between intolerance of uncertainty and gambling urge (Hypothesis 6), as well as that between intolerance of uncertainty and gambling involvement (Hypothesis 7). The conceptual path model with all the hypothesized associations of this study is shown in Figure 1.

## 2. Materials and Methods

### 2.1. Participants and Procedures

The current study involved an anonymous questionnaire survey conducted with a convenience sample of Chinese lottery buyers recruited at public places near lottery sales shops in five different cities (i.e., Chongqing, Leshan, Enshi, Suzhou, and Wenzhou) in mainland China from November 2021 to February 2022. Trained research assistants approached potential participants and explained to them the purposes of the study and their participation rights (e.g., no negative consequences for participation refusal/withdrawal). Eligible and willing participants (i.e., aged 18 and above who had bought lottery tickets in the past year) would be distributed a consent form and an anonymous questionnaire to complete on site. Ethical approval of this study was acquired from the department of psychology of the affiliated university of the corresponding author (reference number: DPSY2021-21). A small monetary incentive (about 1.5 USD on average) was provided to participants who completed and returned the questionnaire. A total of 714 questionnaires was collected, of which, 580 cases (*M_age_* = 34.07, *SD* = 13.36; 50.4% female) were considered valid (i.e., correct answers to two attention test questions) for formal data analysis.

### 2.2. Measures

#### 2.2.1. Intolerance of Uncertainty

The 12-item short version [7] of Freeston et al.’s Intolerance of Uncertainty Scale [46] was used to assess individuals’ negative reactions to uncertainty. The scale has been validated and used in Chinese adult samples [47,48]. Participants answered each item (e.g., “Unforeseen events upset me greatly”) on a 5-point response scale from 1 = *not at all characteristic of me* to 5 = *entirely characteristic of me*. A higher total score indicated a higher level of intolerance of uncertainty. In this study, the Cronbach’s alpha for this scale was 0.82.

#### 2.2.2. Difficulties in Emotion Regulation

In the present study, two subscales of the Chinese version [49] of the Difficulties in Emotion Regulation Scale [30] were used to measure impulse control difficulties (6 items; e.g., “When I’m upset, I lose control over my behaviors”) and limited access to emotion regulation strategies (8 items; e.g., “When I’m upset, I believe that there is nothing I can do to make myself feel better”). All items were answered on a 5-point Likert scale ranging from 1 = *almost never* to 5 = *almost always*. A higher total score of a subscale indicated a higher level of the respective difficulties in emotion regulation. Their Cronbach’s alphas were 0.78 and 0.82, respectively, in this study.

#### 2.2.3. Gambling Urge 

The Chinese version [50] of the 6-item Gambling Urge Scale [23] was used to measure participants’ current gambling urge status. Sample items, such as “Nothing would be better than having a gamble right now”, were answered on a 7-point scale, ranging from 1 = *strongly disagree* to 7 = *strongly agree*. A higher total score indicated a stronger urge to gamble. The Cronbach’s alpha was 0.93 in this study.

#### 2.2.4. Gambling Involvement

With reference to Yakovenko et al. [51], three behavioral indicators were chosen to form a latent factor of gambling involvement in this study: gambling frequency, gambling expenditure, and gambling knowledge acquisition. They were selected from the Behavioral subscales of the Revised Gambling Motives, Attitudes, and Behaviors Inventory which was developed and validated among Chinese gamblers [43,52]. Participants rated their gambling frequency (i.e., “How often I have participated in gambling activities in the past 12 months”) on a 5-point response scale from 1 = *never* to 5 = *always*. They also rated their gambling expenditure (i.e., “I spend lots of money on gambling in the past 12 months”) and acquisition of gambling knowledge (i.e., “I search and investigate various aspects of gambling in order to enhance my chance of winning in the past 12 months”) were answered on a 4-point response scale, ranging from 1 = *never*, 2 = *rarely*, 3 = *sometimes*, to 4 = *often*.

#### 2.2.5. Demographics

The participants were asked to report their gender (1 = *female*, 0 = *male*) and age (years).

### 2.3. Data Analysis

The descriptive and correlation analyses were conducted in SPSS 26.0 [53]. The path model was then tested in the Lavaan package of R with the full information maximum likelihood estimation method, which deals with missing data [54]. According to the recommendation from Kline [55], comparative fit index (CFI; >0.90), root mean square error of approximation (RMSEA; <0.08), standardized root mean square residual (SRMR; <0.08), and Tucker–Lewis index (TLI; >0.90) were used to evaluate whether our hypothesized mediation model had acceptable model fit. If the proposed path model did not fit our data well, modification indexes would be considered and residual covariances might be added in order to improve the model fit. For mediation testing, the total and indirect effects of a variable were estimated with a 95% confidential interval based on the bias-corrected percentile method with 5000 bootstrap samples. Statistical significance was accepted at *p* < 0.05 in all analyses.

## 3. Results

### 3.1. Preliminary Analysis

The questionnaire covered all gambling-related items, i.e., buying lottery tickets as well as other gambling activities such as playing mahjong and poker. In our sample, 11.6% of the participants reported “*often*” or “*always*” on their gambling participation, 6.8% “*sometimes*” or “*often*” spent lots of money on gambling, and 18.9% “*sometimes*” or “*often*” searched for and investigated various aspects of gambling for a higher winning chance in the past year.

The mean, standard deviation, and correlation coefficients of the demographic variables with the gambling variables are shown in Table 1. Age was positively correlated with gambling urge (*r* = 0.29, *p* < 0.001) and the three indicators of gambling involvement (*rs* = 0.15 to 0.28, *ps* < 0.001), while female gender was correlated with a lower level of gambling urge (*r* = −0.21, *p* < 0.001) and gambling involvement (*rs* = −0.13 to −0.16, *ps* < 0.01).

### 3.2. Hypothesis Testing (H1 to H5) by Correlation Analysis

Hypothesis 1 was supported, that intolerance of uncertainty was positively correlated with gambling urge (*r* = 0.11, *p* < 0.01). However, there were no significant bivariate correlations between intolerance of uncertainty and the three individual indicators of gambling involvement (*rs* = −0.05 to 0.06, *ps* = 0.19 to 0.89). Hypothesis 2 was, thus, not supported. On the other hand, both Hypothesis 3 and Hypothesis 4 were supported. Intolerance of uncertainty had a significant and positive correlation with impulse control difficulties (*r* = 0.37, *p* < 0.001) and limited access to emotion regulation strategies (*r* = 0.47, *p* < 0.001), and both impulse control difficulties and limited access to emotion regulation strategies were significantly and positively associated with gambling urge (*rs* = 0.21 to 0.29, *ps* < 0.001). They were also positively correlated with two out of three indicators of gambling involvement (i.e., gambling expenditure and gambling knowledge acquisition; *rs* = 0.10 to 0.21, *ps* < 0.05), thus partially supporting Hypothesis 5.

### 3.3. Hypothesis Testing (H6 and H7) by Path Analysis

**Figure 2:**
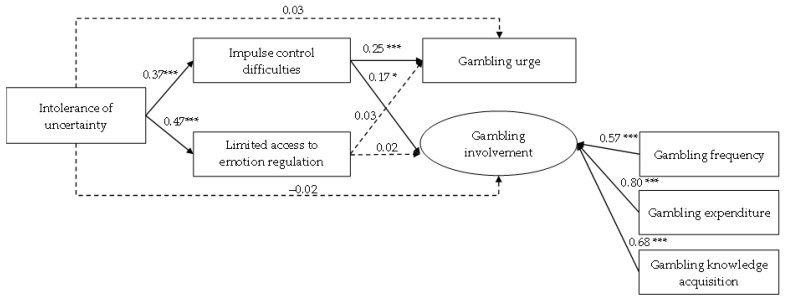
The final path model with standardized estimates. Note: * *p* < 0.05, *** *p* < 0.001. This model has controlled the effects of age and gender on gambling urge and gambling involvement, and the covariance of age and gender. Moreover, the error covariances between impulse control difficulties and limited access to emotion regulation strategies, as well as gambling urge and gambling involvement, were allowed and statistically significant (*p* < 0.001).

After controlling for the effects of age and gender on gambling urge and gambling involvement and allowing the covariation between age and gender, the conceptual path model (Figure 1) was tested by path analysis. Its model fit was poor, χ^2^ (20) = 549.06, CFI = 0.60, TLI = 0.29, RMSEA = 0.21, 90% CI [0.20, 0.23], SRMR = 0.11. According to modification indices, residual covariances between (i) impulse control difficulties and limited access to emotion regulation strategies, as well as (ii) gambling urge and gambling involvement, were allowed to improve the model fit. The modified path model showed a good fit with the data, χ^2^ (18) = 58.20, CFI = 0.97, TLI = 0.94, RMSEA = 0.06, 90% CI [0.05, 0.08], SRMR = 0.04. In the modified path model (Figure 2), all the standardized coefficients of the hypothesized paths were statistically significant, with the exceptions of the direct paths from intolerance of uncertainty to both gambling urge (*β* = 0.03, *p* = 0.48) and involvement (*β* = −0.02, *p* = 0.69), as well as the direct paths from the limited access to emotion regulation strategies to gambling urge (*β* = 0.03, *p* = 0.66) and involvement (*β* = 0.02, *p* = 0.75). Our results suggested that the effect of intolerance of uncertainty to gambling involvement was somehow fully mediated by impulse control difficulties.

Based on the bootstrapping results, the total effect of intolerance of uncertainty on gambling urge and involvement was 0.19 (95% CI [0.04, 0.33]), which was statistically significant and positive. Consistent with the path model, the indirect effect from intolerance of uncertainty to gambling urge was significant via impulse control difficulties (*β* = 0.09, 95% CI [0.05, 0.14]), but not limited access to emotion regulation strategies (*β* = 0.01, 95% CI [−0.04, 0.07]). Similarly, the association between intolerance of uncertainty and gambling involvement was significantly mediated by impulse control difficulties (*β* = 0.06, 95% CI [0.01, 0.12]), but not by limited access to emotion regulation strategies (*β* = 0.01, 95% CI [−0.06, 0.07]). Thus, both Hypotheses 6 and 7 were partially supported.

## 4. Discussion

In China, some gambling activities, such as buying lottery tickets and playing mahjong, are culturally accepted as recreational [3], although people are always advised to restrain to responsible involvement with limited time and wager sizes. This study showed a small but not insignificant percentage of Chinese gamblers engaging in frequent gambling (11.6% for “*often*” or “*always*”), big spending (6.8% for “*sometimes*” or “*often*”), and gambling-related knowledge acquisition for increasing their winning chance (18.9% for “*sometimes*” or “*often*”) during the past year. Our study was also the first to apply the intolerance of uncertainty model to understand whether and how individuals’ disposition (i.e., intolerance of uncertainty) and inabilities (i.e., emotion regulation difficulties) would contribute to both gambling urge and gambling involvement, which were shown to be positively correlated. The findings may help health practitioners to better understand the psychological mechanisms underlying gambling urge and involvement, which may facilitate responsible gambling promotion in the Chinese context.

This study provides empirical evidence for the application of the intolerance of uncertainty model to explain gambling urge and involvement. Although the bivariate correlations of intolerance of uncertainty with individual gambling involvement indicators were non-significant, our multivariate analysis showed that the overall effect of intolerance of uncertainty on gambling urge and involvement was statistically significant and positive in the path model. Our findings enriched the literature of uncertainty intolerance since previous studies examined only its risk effect on either alcohol use motivation [56,57] or problematic technological use in Internet/smartphone [21,22]. In addition, our path model findings showed that the risk effect of intolerance of uncertainty on gambling, which could also be vulnerable to behavioral addiction, was in fact fully mediated by failure or difficulties in one’s emotion regulation.

Consistent with the intolerance of uncertainty model, intolerance of uncertainty was the risk factor of not only gambling urge/involvement but also emotional regulation difficulties (i.e., impulse control difficulties and limited access to emotion regulation strategies). These findings were in line with the previous ones that individuals who were more intolerant to uncertainty suffered from greater difficulties to control their impulsive behaviors or adopt adaptive emotion regulation strategies [12,26,27]. The results of our path analysis further suggested that gamblers’ difficulties in emotion regulation (mainly via the impulse control route) could be attributed to intolerance of uncertainty, increasing their urge and behaviors related to gambling. Since addictive behaviors such as gambling are often adopted for coping with negative emotions such as fear, anxiety, and worry [45,58,59], which are probably heightened with the trait of uncertainty intolerance, difficulties of emotion regulation in gamblers must be identified and intervened via for example skill training [60] in promotion of responsible gambling. Given the full mediating role of emotion regulation difficulties in the relationship between uncertainty intolerance and gambling, future studies should also address the knowledge gap by examining how uncertainty intolerance weakens one’s emotional regulation functioning via various potential emotion-cognitive processes (e.g., increasing emotional loads and attentional bias) in order to provide further insights for effective interventions.

Despite the high correlation between the two types of difficulties of emotional regulation [30,49,61], in our study, only impulse control difficulties significantly mediated the effects of intolerance of uncertainty on gambling urge and involvement in the path model. It suggested that impulse control difficulties cast a more salient role than limited access to emotion regulation strategies on the association between intolerance of uncertainty and gambling. This is consistent with Marchica et al.’s findings [62] that impulse control difficulties, and not limited access to emotion regulation strategies, contributed more to GD vulnerability in emerging adult gamblers. A recent study of Chinese university students [63] similarly found that among the two components of self-regulation, only impulse control and not goal setting made significant contribution to proneness of behavioral addictions (assessed by measures for smartphone addiction and Internet gaming disorder). Future research could empirically evaluate whether impulse control (difficulty) is the most important risk factor, as compared to other self-regulation components, in gambling across ages and cultural groups.

The present study is not without limitations. First, we recruited the participants by convenience sampling and this method may limit the generalizability of our findings to the overall gambler community. Future research is recommended to recruit a more representative group of gamblers with a stratified sampling via both online and offline gambling sites. Second, the correlations between intolerance of uncertainty and gambling variables were relatively weak in our sample, who were recruited near lottery sales shops. Given intolerance of uncertainty model was generally developed to explain mental disorders such as generalized anxiety disorder, we wonder whether the correlation between intolerance of uncertainty with other gambling variables (such as symptoms of gambling disorder) would be stronger among high-risk or disordered gamblers. Therefore, further research is also warranted to explore the relationship between intolerance of uncertainty and different gambling variables (not only gambling urge and involvement but also symptoms of gambling disorder) across gambler types (e.g., casino gamblers vs. lottery buyers; clinical sample vs. community sample). Third, all variables were measured by self-reported items, which might be biased due to social desirability and self-serving cognitions. Future studies could incorporate multi-modal assessments on gambling risk status (e.g., potential neural markers of GD and clinical diagnosis made by psychiatrists). Last, but not least, the present study used a cross-sectional design, which does not allow testing the predictive effects of intolerance of uncertainty and emotion regulation on gambling urge and involvement. Longitudinal studies are warranted to further investigate the temporal relationships among the variables.

## 5. Conclusions

The present study is the first to test the applicability of the intolerance of uncertainty model to gambling urge and involvement. In line with previous studies on intolerance of uncertainty and emotion regulation difficulties, our findings showed that a higher level of intolerance of uncertainty contributed more difficulties in emotion regulation. In addition, impulse control difficulties, and not limited access to emotion regulation strategies, explained how higher levels of intolerance of uncertainty contribute to greater gambling urge and higher gambling involvement. Our findings identified impulse control difficulties as the most salient risk factor for gambling urge and involvement, and, hence, this specific type of emotion regulation difficulties may be targeted in future programs for responsible gambling promotion.

## Figures and Tables

**Table 1:** Descriptive statistics and inter-correlations of all variables.

	*M*	*SD*	1	2	3	4	5	6	7	8
1. Intolerance of uncertainty	34.41	6.94	--							
2. Impulse control difficulties	13.55	3.89	0.37 ***	--						
3. Limited access to emotion regulation strategies	19.38	5.32	0.47 ***	0.74 ***	--					
4. Gambling urge	13.52	7.19	0.11 **	0.29 ***	0.21 ***	--				
5. Gambling frequency	2.56	0.79	−0.05	−0.01	0.01	0.30 ***	--			
6. Gambling expenditure	1.44	0.65	0.01	0.21 ***	0.12 **	0.44 ***	0.38 ***	--		
7. Gambling knowledge acquisition	1.74	0.87	0.06	0.15 ***	0.10 *	0.45 ***	0.47 ***	0.53 ***	--	
8. Age	34.07	13.36	−0.06	0.04	−0.02	0.29 ***	0.26 ***	0.15 ***	0.28 ***	--
9. Gender ^#^	--	--	0.07	−0.03	0.07	−0.21 ***	−0.13 **	−0.13 **	−0.16 ***	−0.34 ***

Note: * *p* < 0.05, ** *p* < 0.01, *** *p* < 0.001. ^#^ Binomial variable: 1 = *Female*, 0 = *Male*. 1 = Intolerance of uncertainty, 2 = Impulse control difficulties, 3 = Limited access to emotion regulation strategies, 4 = Gambling urge, 5 = I Gambling frequency, 6 = Gambling expenditure, 7 = Gambling knowledge acquisition, 8 = Age, 9 = Gender.

## Data Availability

The datasets generated during and/or analyzed in the current study are available from the corresponding author on reasonable request.
